# Supplementary material for: Gate-based quantum computing for protein design
Source: PLoS Comput Biol. 2023 Apr 12;19(4):e1011033. doi: 10.1371/journal.pcbi.1011033 (PMC10124842; doi:10.1371/journal.pcbi.1011033)
Supplement: S1 Appendix — (PDF) [file pcbi.1011033.s001.pdf]

S1. Number of required qubits in the circuit and the general algorithm

In this section, we describe how to estimate the m value, as well as the total number of qubits for different circuits. To do so, in each circuit, we first need to estimate the largest possible positive value and smallest possible negative value. This estimation is based on the Hamiltonian and the energy table associated with the system of study.

Each value in the circuit is represented by a set of qubits shown in Fig. S1-A. One can simply check that using n qubits, we can show 2^n numbers. For example, by using 6 qubits, we can show 64 numbers, i.e., 0 to 63. However, if we need to show both negative and positive numbers, the most left qubit is devoted to the sign, as shown in Fig. S1-A. Thus, we can show numbers from −32 to +31 using six qubits.

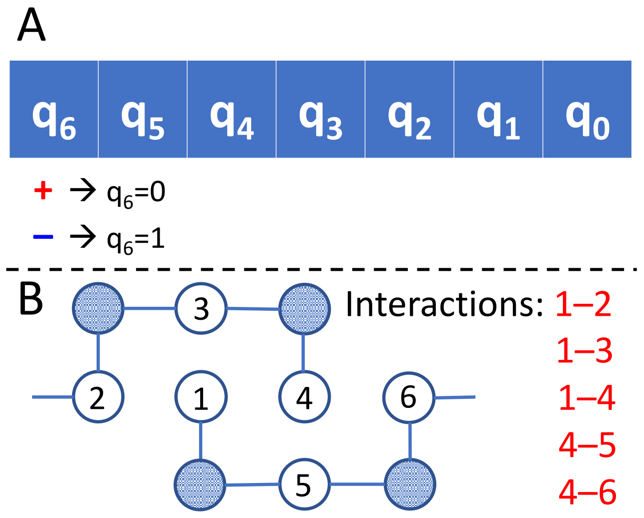

FIG. S1. A) Schematic representation of 7 qubits. B) Schematic representation of a system with 6 interacting residues and 5 interactions.

Here, we focus on a system in the SP model with six designable sites and five interactions, as shown in Fig. S1-B. Using the energy table in the Fig. 2-A of the main text (and Fig. S5- B), the largest energy value is +4, and the smallest is −4. Since we have five interactions, the extremes of the total energy of a set can be −20 and +20. Now, if we subtract these numbers from $E_{th} = -19$, the range of values in the circuit will be from +39 to −1. Based on the calculations described earlier, using six qubits to represent numbers is insufficient for +39 to −1 range, as it can show −32 to +31 in a standard implementation. However, using seven qubits can represent numbers in the −64 to +63 range that covers our +39 to −1values.

In our circuits, $q = n + \#\_work\_qubits$ (Fig. S2-A). To calculating the total number of qubits required for the circuit in Fig. S1-B, we first calculate the number of *n* qubits. Since, *n = 3 × s*, and *s=6* for 6 designable sites, thus *n = 18* (Fig. S2-B).
The $\#\_work\_qubits$, which is $2m + 1$, is describe as following:

1. We allocate four qubits sets, one with 18 (generally $3 \times s$) qubits labelled as "var_qubits", one with a single qubit (required by the adder) labelled as "o_qubit"

and two sets, each with 7 (generally *m*) qubits, labelled as “a_qubits” and “b_qubits”.
2. The energy of the interaction 1 − 2 is put in the a_qubits.
3. The energy of the interaction 1 − 3 is put in the b_qubits.
4. The value in the a_qubits is added to b_qubits and put into a_qubits (using quantum ripple adder).
5. The energy of the interaction 1 − 3 is cleaned from the b_qubits (to inter the next value).
6. The energy of the interaction 1 − 4 is put in the b_qubits.
7. The value in the a_qubits is added to b_qubits and put into a_qubits (now we have 1-2, 1-3 and 1-4 adde together).
8. The energy of the interaction 1 − 4 is cleaned from the b_qubits.
9. The energy of the interaction 4 − 5 is put in the b_qubits.
10. The value in the a_qubits is added to b_qubits and put into a_qubits.
11. The energy of the interaction 4 − 5 is cleaned from the b_qubits.
12. The energy of the interaction 4 − 6 is put in the b_qubits.
13. The value in the a_qubits is added to b qubits and put into a_qubits.

Now, we have added all energies in the configuration. We only need 18 qubits for n and 7 + 7 qubits for the a_qubits + b_qubits and 1 qubit for the o_qubit, in total 33 qubits.

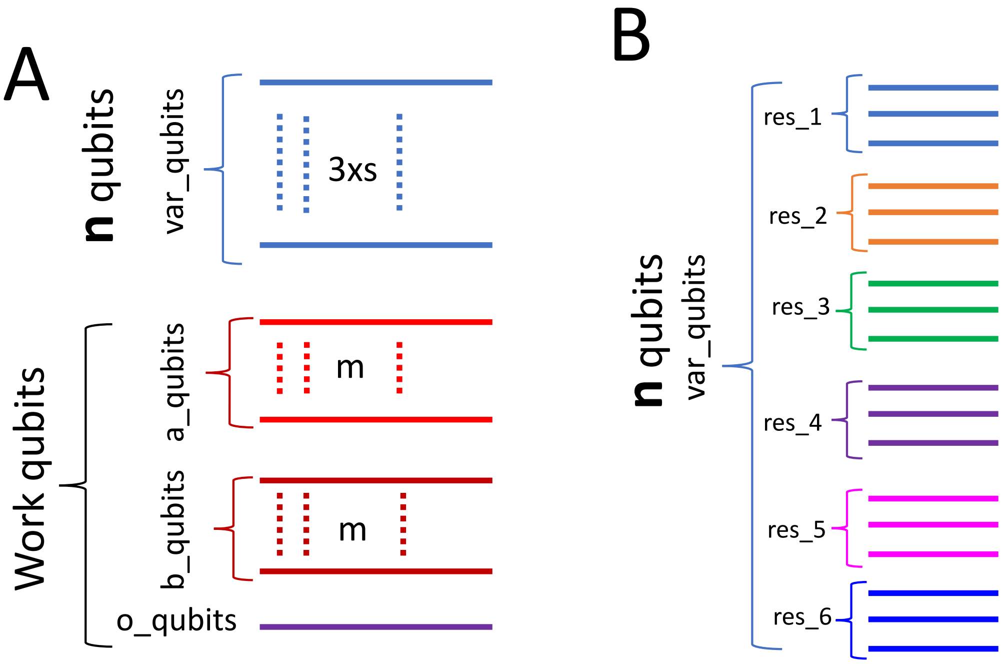

FIG. S2. General representation of qubits in our algorithms. A) qubits representation in the whole circuit. B) *n* qubits for 6 designable site/residues system.
