# Supplementary material for: Gate-based quantum computing for protein design
Source: PLoS Comput Biol. 2023 Apr 12;19(4):e1011033. doi: 10.1371/journal.pcbi.1011033 (PMC10124842; doi:10.1371/journal.pcbi.1011033)
Supplement: S2 Appendix — (PDF) [file pcbi.1011033.s002.pdf]

S2. Explicit results of each system

Answer states of all systems studied in the main paper are provided in Tables S1, S2 and S3.

TABLE S1. Answers for all systems studied in the SP model.

s=2		s=3		s=6,i=3		
E_{th}=-3	E_{th}=-2	E_{th}=-7	E_{th}=-5	E_{th}=-11	E_{th}=-10	
Seq(2 – 1)		Seq(3 – 2 – 1)		Seq(6 – 5 – 4 – 3 – 2 – 1)		
Pos-Neg<br>Neg-Pos	H1-H1<br>Pol1-Pol1<br>Pol2-Pol2<br>Pos-Neg<br>Neg-Pos	Pos-Pos-Neg<br>Neg-Neg-Pos	H1-H1-H1<br>Pos-Pos-Neg<br>Neg-Neg-Pos<br>Pol1-Pol1-Pol1<br>Pol2-Pol2-Pol2	Pos-Neg-Pos-Neg-Pos-Neg<br>Pos-Neg-Pos-Neg-Neg-Pos<br>Pos-Neg-Neg-Pos-Pos-Neg<br>Pos-Neg-Neg-Pos-Neg-Pos<br>Neg-Pos-Pos-Neg-Pos-Neg<br>Neg-Pos-Pos-Neg-Neg-Pos<br>Neg-Pos-Neg-Pos-Pos-Neg<br>Neg-Pos-Neg-Pos-Neg-Pos	Pol1-Pol1-Pos-Neg-Pos-Neg<br>Pol1-Pol1-Pos-Neg-Neg-Pos<br>Pol1-Pol1-Neg-Pos-Pos-Neg<br>Pol1-Pol1-Neg-Pos-Neg-Pos<br>Pol2-Pol2-Pos-Neg-Pos-Neg<br>Pol2-Pol2-Pos-Neg-Neg-Pos<br>Pol2-Pol2-Neg-Pos-Pos-Neg<br>Pol2-Pol2-Neg-Pos-Neg-Pos<br>Pos-Neg-H1-H1-Pos-Neg<br>Pos-Neg-H1-H1-Neg-Pos<br>Pos-Neg-Pol1-Pol1-Pos-Neg<br>Pos-Neg-Pol1-Pol1-Neg-Pos<br>Pos-Neg-Pol2-Pol2-Pos-Neg<br>Pos-Neg-Pol2-Pol2-Neg-Pos<br>Pos-Neg-Pos-Neg-H1-H1<br>Pos-Neg-Pos-Neg-Pol1-Pol1<br>Pos-Neg-Pos-Neg-Pol2-Pol2<br>Pos-Neg-Pos-Neg-Pos-Neg<br>Pos-Neg-Pos-Neg-Neg-Pos<br>Pos-Neg-Neg-Pos-H1-H1<br>Pos-Neg-Neg-Pos-Pol1-Pol1<br>Pos-Neg-Neg-Pos-Pol2-Pol2	Pos-Neg-Neg-Pos-Pos-Neg<br>Pos-Neg-Neg-Pos-Neg-Pos<br>Neg-Pos-H1-H1-Pos-Neg<br>Neg-Pos-H1-H1-Neg-Pos<br>Neg-Pos-Pol1-Pol1-Pos-Neg<br>Neg-Pos-Pol1-Pol1-Neg-Pos<br>Neg-Pos-Pol2-Pol2-Pos-Neg<br>Neg-Pos-Pol2-Pol2-Neg-Pos<br>Neg-Pos-Pos-Neg-H1-H1<br>Neg-Pos-Pos-Neg-Pol1-Pol1<br>Neg-Pos-Pos-Neg-Pol2-Pol2<br>Neg-Pos-Pos-Neg-Pos-Neg<br>Neg-Pos-Pos-Neg-Neg-Pos<br>Neg-Pos-Neg-Pos-H1-H1<br>Neg-Pos-Neg-Pos-Pol1-Pol1<br>Neg-Pos-Neg-Pos-Pol2-Pol2<br>Neg-Pos-Neg-Pos-Pos-Neg<br>Neg-Pos-Neg-Pos-Neg-Pos<br>H1-H1-Pos-Neg-Pos-Neg<br>H1-H1-Pos-Neg-Neg-Pos<br>H1-H1-Neg-Pos-Pos-Neg<br>H1-H1-Neg-Pos-Neg-Pos

s=6,i=4			s=6,i=5	
E_{th}=-15	E_{th}=-13		E_{th}=-19	E_{th}=-16
Seq(6 – 5 – 4 – 3 – 2 – 1)			Seq(6 – 5 – 4 – 3 – 2 – 1)	
Pos-Pos-Neg-Pos-Pos-Neg<br>Pos-Pos-Neg-Neg-Neg-Pos<br>Neg-Neg-Pos-Pos-Pos-Neg<br>Neg-Neg-Pos-Neg-Neg-Pos	H1-H1-H1-Pos-Pos-Neg<br>Pol1-Pol1-Pol1-Pos-Pos-Neg<br>Pol1-Pol1-Pol1-Neg-Neg-Pos<br>H1-H1-H1-Neg-Neg-Pos<br>Pol2-Pol2-Pol2-Pos-Pos-Neg<br>Pol2-Pol2-Pol2-Neg-Neg-Pos<br>Pos-Pos-Neg-H1-H1-H1<br>Pos-Pos-Neg-Pol1-Pol1-Pol1	Pos-Pos-Neg-Pol2-Pol2-Pol2<br>Pos-Pos-Neg-Pos-Pos-Neg<br>Pos-Pos-Neg-Neg-Neg-Pos<br>Neg-Neg-Pos-H1-H1-H1<br>Neg-Neg-Pos-Pol1-Pol1-Pol1<br>Neg-Neg-Pos-Pol2-Pol2-Pol2<br>Neg-Neg-Pos-Pos-Pos-Neg<br>Neg-Neg-Pos-Neg-Neg-Pos	Pos-Pos-Neg-Neg-Neg-Pos<br>Neg-Neg-Pos-Pos-Pos-Neg	Pol1-Neg-Pos-Pos-Pos-Neg<br>Pos-Pos-Neg-Pol1-Neg-Pos<br>Pos-Pos-Neg-Neg-Pol1-Pos<br>Pos-Pos-Neg-Neg-Neg-Pos<br>Neg-Pol1-Pos-Pos-Pos-Neg<br>Neg-Neg-Pos-Pos-Pos-Neg

TABLE S2. Answers for all systems studied in the default MR model.

s=2				s=3			s=3 (symmetric)
E_{th}=95%E_{max}	E_{th}=85%E_{max}	E_{th}=80%E_{max}	E_{th}=70%E_{max}	E_{th}=80%E_{max}	E_{th}=70%E_{max}	E_{th}=50%E_{max}	E_{th}=50%E_{max}
Seq(2 – 1)				Seq(3 – 2 – 1)			
Pos-Neg<br>Neg-Pos	Pol1-Pol1<br>Pos-Neg<br>Neg-Pos	Pol1-Pol1<br>Pol2-Pol2<br>Pos-Neg<br>Neg-Pos	H1-H1<br>Pol1-Pol1<br>Pol2-Pol2<br>Pos-Neg<br>Neg-Pos	Pol1-Pol1-Pol1	H1-H1-H1<br>Pol1-Pol1-Pol1<br>Pol2-Pol2-Pol2	H1-H1-H1<br>H1-H1-H2<br>Pos-Neg-Pol1<br>Pos-Neg-Pos<br>Pos-Neg-Neg<br>Pos-Neg-X2<br>Neg-Pos-Pol1<br>Neg-Pos-Neg<br>Neg-Pos-X2<br>Pol1-Pol1-Pol1<br>Pol1-Pol1-Pol2<br>Pol1-Pol1-Pos<br>Pol1-Pol1-Neg<br>Pol1-Pol2-Pol1<br>Pol1-Pol2-Pol2<br>Pol2-Pol1-Pol1<br>Pol2-Pol1-Pol2<br>Pol2-Pol2-Pol1<br>Pol2-Pol2-Pol2	H1-H1-H1<br>H2-H2-H2<br>Pol1-Pol1-Pol1<br>Pol1-Pol1-Pol2<br>Pol1-Pol2-Pol1<br>Pol1-Pol2-Pol2<br>Pol2-Pol1-Pol1<br>Pol2-Pol1-Pol2<br>Pol2-Pol2-Pol1<br>Pol2-Pol2-Pol2

TABLE S3. Answers for all systems studied in the MR-MP model.

s=2 (MP)				s=3 (MP)	
E_{th}=95%E_{max}	E_{th}=85%E_{max}	E_{th}=80%E_{max}	E_{th}=70%E_{max}	E_{th}=80%E_{max}	E_{th}=70%E_{max}
Seq(2 – 1)				Seq(3 – 2 – 1)	
Pos-Neg<br>Neg-Pos	Pol1-Pol1<br>Pos-Neg<br>Neg-Pos	Pol1-Pol1<br>Pol2-Pol2<br>Pos-Neg<br>Neg-Pos	H1-H1<br>Pol1-Pol1<br>Pol2-Pol2<br>Pos-Neg<br>Neg-Pos	Pol1-Pol1-Pol1<br>Pol2-Pol2-Pol2	H1-H1-H1<br>Pol1-Pol1-Pol1<br>Pol2-Pol2-Pol2
