# Supplementary material for: Gate-based quantum computing for protein design
Source: PLoS Comput Biol. 2023 Apr 12;19(4):e1011033. doi: 10.1371/journal.pcbi.1011033 (PMC10124842; doi:10.1371/journal.pcbi.1011033)
Supplement: S3 Appendix — (PDF) [file pcbi.1011033.s003.pdf]

S3. Computational costs of the simulations on conventional computers

Since we use quantum computer simulators to study our circuits, we are restricted by the limitations of the conventional computers, i.e., the amount of RAM required to simulate a system and how long the simulation will take in real-time (CPU time) to run. Fig. S3 shows the changes in the amount of RAM and CPU time required to simulate different systems as a function of R/R_{max}. For all data sets, increasing the number of iterations increases both RAM amount and CPU time.

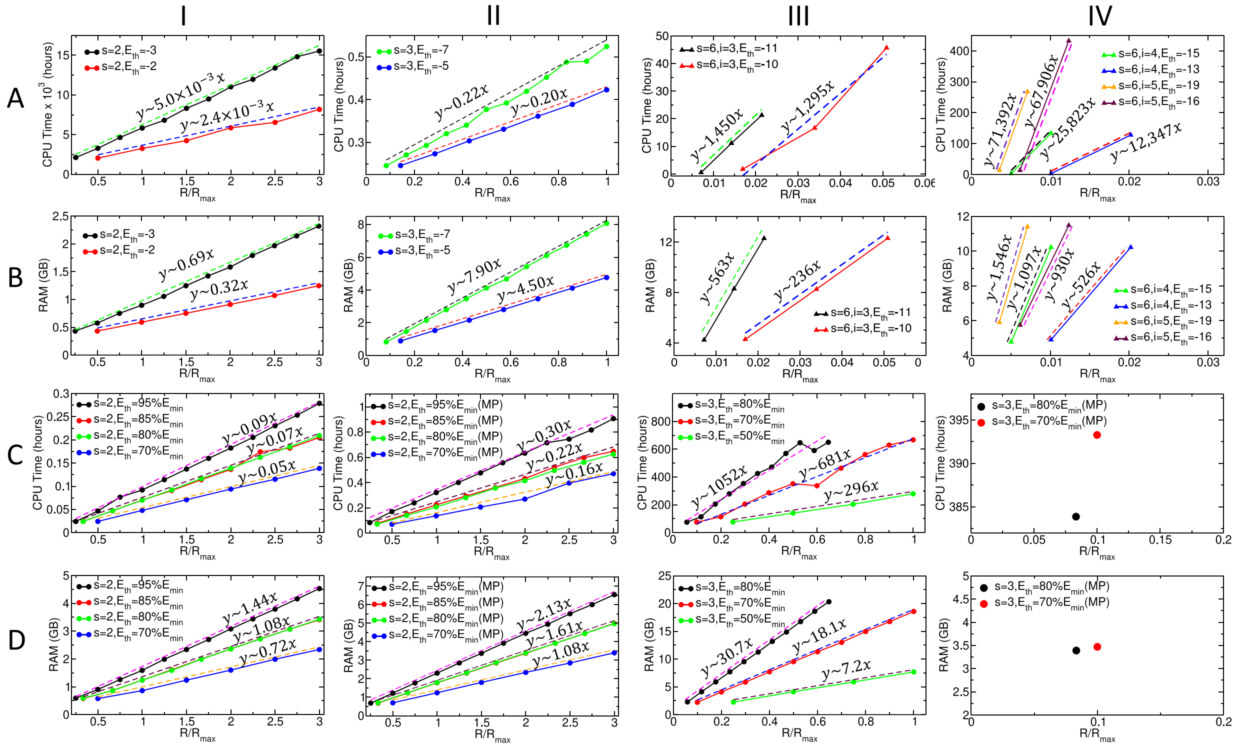

FIG. S3. Change in the cost of simulation as a function of normalized number of iterations for systems in the SP and MR models. Data in rows A) and B) shows the CPU time and the RAM usage for systems in the SP model, respectively. Data in rows C) and D) shows the CPU time and the RAM usage for systems in the MR model, respectively. In rows C) and D) data for the MR-MP are presented in columns II and IV. In all panels, the dashed lines represent the fit to the curves.

Based on the data from circuits with two and three designable sites in the SP model and circuits with two designable sites in the MR model (both default MR and MR-MP) represented in columns I and II in Fig. S3, we can see that the RAM amount and the CPU time are linear functions of R/R_{max}. This linear behaviour is expected as increasing the number of iterations increases the number of times the oracle and the diffuser are called in the algorithm. Thus, since the simulations run on conventional computational resources, increasing the amount of computation linearly with R increases the cost of computation linearly. Expecting a similar linear behaviour, we predict the resources required to simulate the larger circuits that have few actual data points in columns III and IV in Fig. S3. Note that, for the systems with two designable sites in both SP and MR models, the x-axis is extended to $R/R_{max}=3$ to have more data points on the curves to fits.

These results show that circuits in the SP model with two and three designable sites, as well as the ones in the MR model with two designable sites, shown in columns I and II of Fig. S3, could run with $R = R_{max}$ on resources available on a regular laptop device as the required RAM amount is ~8 GB and the maximum CPU time is within an hour time range. However, for the rest of the systems, shown in columns III and IV of Fig. S3, even running the first few iterations takes days of simulation and more than 10 GB of RAM in some cases. In these circuits, running the simulation with $R/R_{max} = 1$ requires significant computational resources and simulation times. As an example, the $s = 6$, $i = 5$, $E_{th} = -19$ system would need ~1.5 TB of RAM and ~71,392 hours (~8.1 years) of CPU time to simulate with $R = R_{max}$ iterations (Fig. S3-A-IV and B-IV).

In the MR model (both the default and MR-MP), the computational resources required to simulate the $s = 2$, $E_{th} = 85\%E_{min}$ and the $s = 2$, $E_{th} = 80\%E_{min}$ systems are almost identical (Fig. S3-C-I and D-I, and C-II and D-II). These results motivated us to study the changes in the CPU time, and the RAM usage as a function of R (instead of R/R_{max}) for all systems in Fig. S3, which is provided in Fig. S4 and will be discussed in more details later in this section. Based on these results, for systems with the same number of designable sites and interactions, the computational resources required to simulate the circuits are almost identical for all E_{th} values, which indicates that the usage of computational resources is independent of the E_{th}.

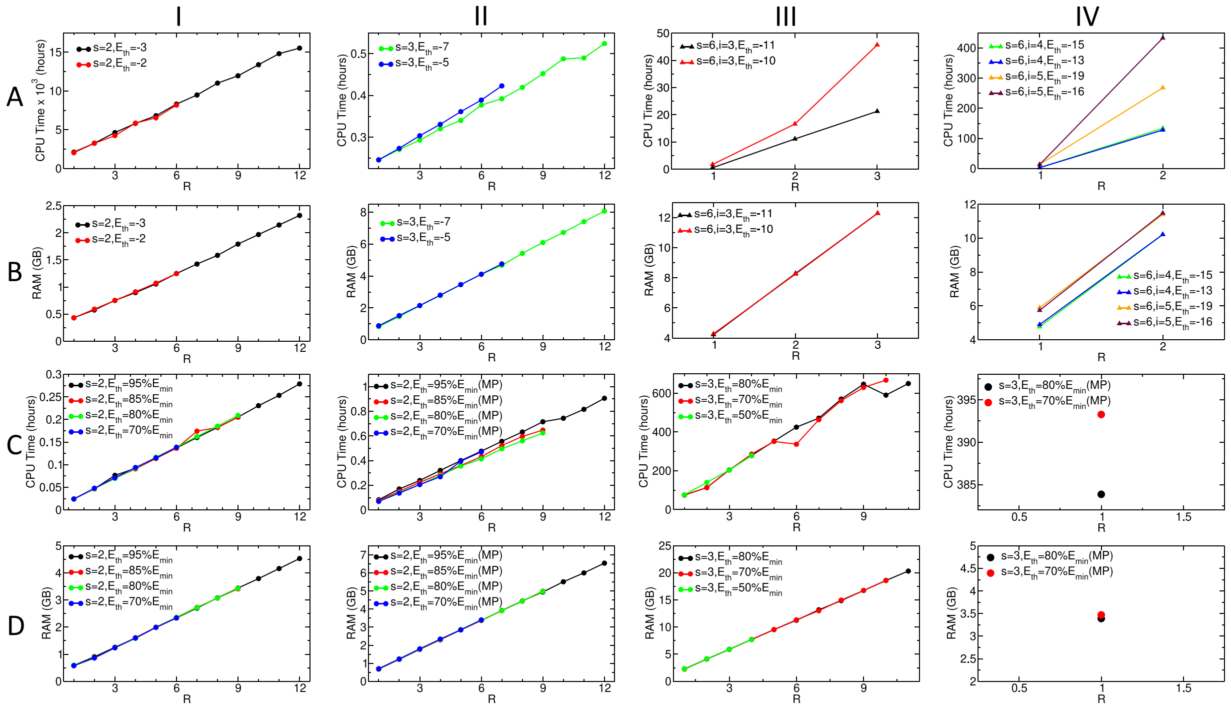

FIG. S4. Change in the cost of simulation as a function of R for systems in the SP and MR models. Data in rows A) and B) shows the CPU time and RAM usage for systems in the SP model, respectively. Data in rows C) and D) shows the CPU time and RAM usage for systems in the MR model, respectively. In rows C) and D) data for default MR are presented in columns I and III, and for the MR-MP in columns II and IV. Only one data point for systems in C-IV and D-IV are available in our simulations. In all panels, the dashed lines represent the fit to the curves.

Moreover, for similar systems in the SP and the MR models, i.e., the $s = 2, E_{th} = -3$ and $s = 2, E_{th} = 95\%E_{min}$ circuits, as well as, the $s = 2, E_{th} = -2$ and the $s = 2, E_{th} = 70\%E_{min}$ circuits, we can see that the CPU time is more than 18 times longer for the MR model systems (Figure S3-A-I and C-I). Also, the RAM usage in the MR model systems is approximately twice the ones in the SP model (Figure S3-B-I and D-I). These differences are expected as the circuits in the MR model are more complex than those in the SP model.

Similarly, comparing the results between the equivalent systems in the two designable site circuits in the MR model, i.e., the default MR and the MR-MP, show that the system with more precision requires ~3.3 times longer CPU times and ~1.5 times more RAM (Figure S3-C-I, C-II, D-I, and D-II). Thus, increasing the precision in a simulation increases the computational cost for similar systems in the MR and the MR-MP models. This is expected since to increase the precision, more qubits are needed in the circuit (Table I in the main text).

Figure S4 reports the CPU time and RAM usage of the system studied as a function of R. In most panels, the curves are overlapping, showing similar resource usage as a function of iteration. However, in some cases, some data points have different values, which do not fit the general trends of curves in similar data sets. This only occurs in the CPU time plots and it is due to different properties of CPUs in the Cedar cluster, which we run our jobs on it. Nevertheless, these differences in the CPU times do not affect the generality of our conclusions in the main text. In case of the $s=6, i=5, E_{th}=-19$ in the SP model, adjusting the current CPU time with the one in a faster CPU device could lead to ~36,500 hours (instead of 71,392 hours), which is around 4.5 years and still unachievable.
