# Supplementary material for: Gate-based quantum computing for protein design
Source: PLoS Comput Biol. 2023 Apr 12;19(4):e1011033. doi: 10.1371/journal.pcbi.1011033 (PMC10124842; doi:10.1371/journal.pcbi.1011033)
Supplement: S4 Appendix — (PDF) [file pcbi.1011033.s004.pdf]

S4. Role of number of iterations

Figure S5 represents the change in the probability of finding answer states as a function of the number of iterations for selected systems in the SP and MR models. We can see that the probability curves follow the $\sim sin^2((aR + b)/c)$ patterns, where a, b and c are constant values. This pattern is an expected behaviour of Grover's algorithm and is more evident and smoother for the system with three designable sites in the SP model (Fig. S5-B). By increasing the number of iterations, the probability of finding answer states increases. However, after reaching the R_{max} (Eq. 8 in the main paper), which is the first peak value in all curves in Fig. S5, the probability of finding answer states decreases.

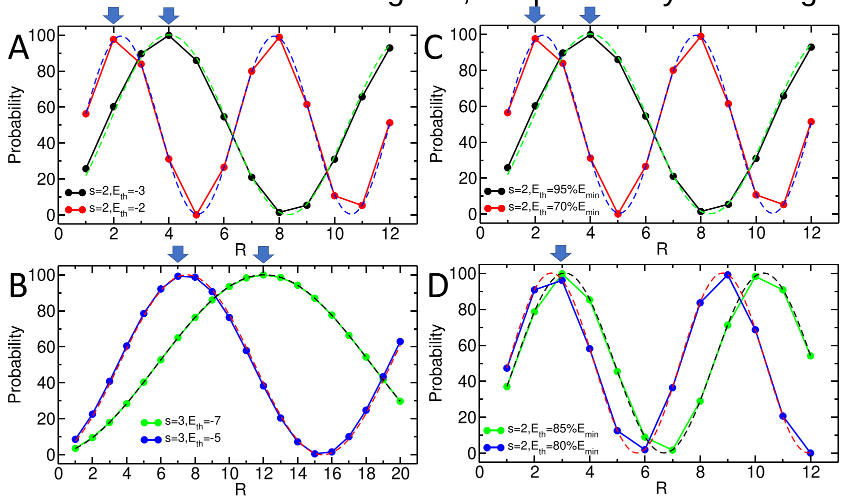

FIG. S5. Probability of finding answer states as a function of the number of iterations R in different systems. The SP model with: A) Two designable sites; B) Three designable sites. The MR model with two designable sites with: C) Eth = 95%Emin and Eth = 70%Emin; D) Eth = 85%Emin and Eth = 80%Emin. The dashed lines show the sin2((aR + b)/c) curves fitted to each data set. The first local maximum on each curve is pointed by the blue arrow.

Moreover, the curves for the systems with two designable sites in the SP model with $E_{th} = -3$ and $E_{th} = -2$ (Fig. S5-A) and the ones in the MR model with $E_{th} = 95\%E_{min}$ and $E_{th} = 70\%E_{min}$ (Fig. S5-C) are almost identical. As discussed earlier, this behaviour is expected for systems with the same total number of states N and the same number of answer states M.

Nevertheless, two systems can have the same R_{max} value, but different patterns for the probability of finding the answer states as a function of R. For example, for two systems in the MR model, i.e., $s = 2, E_{th} = 85\%E_{min}$ and $s = 2, E_{th} = 80\%E_{min}$ systems, with three and four answer states respectively, the R_{max} value is 3 (Fig. S5-D). However, despite having the same R_{max}, the curves for the two systems differ significantly, where even their second peaks occur in different R values. This result shows that despite having the same N states and the same R_{max} value, if two systems have different N/M, their probability curves will have different patterns.
