# Supplementary material for: Gate-based quantum computing for protein design
Source: PLoS Comput Biol. 2023 Apr 12;19(4):e1011033. doi: 10.1371/journal.pcbi.1011033 (PMC10124842; doi:10.1371/journal.pcbi.1011033)
Supplement: S5 Appendix — (PDF) [file pcbi.1011033.s005.pdf]

## S5. Computational costs in the circuits

## I: quantum circuit

To find the number of gates for each step of Grover's algorithm implemented in our circuits, deep knowledge of each step is required. To this end, we investigate the SP model circuits.

The number of computations in the initialization step ($\#ofQ_{init.}$) is equivalent of number of *H*-gates used, which is *n* (Fig. S7-C)*.* Thus, $\#ofQ_{init.}{\sim}\mathcal{O}(n) = \mathcal{O}(\log_2(N))$.

Next, we need to calculate the cost of computation for the oracle, i.e., $\#ofQ_{orcl.}$. Here, the computation cost consists of introducing energies to the work qubit, adding them up and negating the answer states, and finally cleaning up the gates (Fig. 1-b in the main text).

To introduce energies, we use the *6-control-1-NOT* gates represented in Fig. S7-C. The maximum required *6-control-1-NOT* gates will be used to represent value of –1 in the circuit, which requires applying 7 (as *m=7*) of the *6-control-1-NOT* gates. For the other values in the pair-wise energy table, lesser number of the gates are required. However, to be on the safe side, we will assume all values in the energy table are –1. For each value, we require some *X* gates as shown in Fig. S7-C. Similarly, we consider the maximum number of *X* gates that is required, which is 12 (as $2 \times 2 \times \lceil log_2(total\ number\ of\ residues)\rceil$, which is a constant for our circuits) gates for the *H1-H1* pair. Thus, introduce energy values each time we require

$$8 \times 8 \times [m \times (6CX) + 12 \times \mathrm{X}] \text{ (Eq. S1)}$$

gates, where *6CX* represents the *6-control-1-NOT* gates. From Eq. 1, Eq. 2 and Eq. 3 in the main text, we have that $m{\sim}\,\mathcal{O}(\log_2(\log_2 N))$, which simplifies the Eq. S1 to $\sim\mathcal{O}(\log_2(\log_2 N)) \times (6CX)$. It is not possible for current quantum computers to implement the *6-control-1-NOT* gates as a single gate, which is a task for future quantum computers with fully connected qubit mappings. From the Nielsen-Chuang book on "Quantum computation and quantum information", we have that an n-*contol-1-X* gate can be decomposed into *2(n–1) CCX* gates, where each *CCX* can be decomposed into 15 single and CX gates. Thus, Eq. S1 with decomposition of $6CX$ simplifies to $\sim\mathcal{O}(\log_2(\log_2 N)) \times \mathcal{O}(\log_2 N) \approx \mathcal{O}(\log_2(\log_2(N)) \times \log_2(N))$. Thus, the number of computations required for introducing the energies to the circuit is ${\sim}\mathcal{O}(\log_2(\log_2(N)) \times \log_2(N))$. Note that for a fully connected mapping, this number will be ${\sim}\mathcal{O}(\log_2(\log_2(N))$ instead of ${\sim}\mathcal{O}(\log_2(\log_2(N)) \times \log_2(N))$. For the *SP,s=2* system with any $E_{th}$ value, the introducing the energy is done once (description is provided in the algorithm part of section I of the supplementary data). However, for each bond *i* in the structure, we need to introduce the energies for them. However, for *s>2* (i.e., *i>1*) we need to clean qubits from values of the previous bond (by re-applying the gates used to introduce them in the first place) and add the new bond's energy values (algorithm part of section I of the supplementary data). This requires applying the introducing the energy step $2i - 1$ times, where $i{\sim}\mathcal{O}(\log_2(N))$. Thus, the total cost of the introducing energies at worst case scenario will be ${\sim}\mathcal{O}(\log_2(\log_2(N)) \times \log_2(N)) \times \mathcal{O}(\log_2(N)) \approx \mathcal{O}[\log_2(\log_2(N)) \times (\log_2(N))^2]$.

Then, there is the cost of adders, which is discussed briefly in the main text and changes as $\sim\mathcal{O}(m) \approx \mathcal{O}(\log_2((\log_2(N)))$. Thus, to add the energies together, each time an $\sim\mathcal{O}(\log_2((\log_2(N)))$ computation is implemented in the circuit. Since energies of each interaction *i* is added to find the $E_{tot}$, the computation cost is $\sim\mathcal{O}(\log_2(\log_2(N)) \times i \approx \mathcal{O}(\log_2((\log_2(N))) \times \mathcal{O}(\log_2(N)) \approx \mathcal{O}(\log_2((\log_2(N)) \times \log_2(N))$.

After summing the energies between all designable sites, circuits subtract the $E_{tot}$ from the $E_{th}$, which adds another $\sim\mathcal{O}(\log_2(\log_2(N)))$ computation to the system (same as adder function).

For the negation, a single *CZ* gate is used for negating the answer states. Finally, the work qubits are cleaned by applying the inverse of the functions initially implemented, meaning that all computations applied before the negating process will duplicated.

Thus, the total number of computation used in the oracle is $2 \times [cost\_energy\_introduction + cost\_adders\_E_{tot} + cost\_subtract\_E_{th} + cost\_CZ\_gate]$. Using the values provided in previous discussions in this section, the cost of the oracle will be
$\sim\mathcal{O}\ [(\log_2(\log_2(N)) \times (\log_2(N))^2) + (\log_2((\log_2(N)) \times \log_2(N)) + \log_2(\log_2(N))]$,
or

$$\sim\mathcal{O}[\log_2(\log_2(N)) \times ((\log_2(N))^2 + \log_2(N) + 1)]$$

Finally, we should consider the cost of computation in the diffuser step ($\#ofQ_{diff.}$). From the Nielsen-Chuang book on "Quantum computation and quantum information", we can see that the diffuser is composed of *2(n+1) H*-gates, *2n X*-gates and one *(n-1)-control-1-NOT* gate. The *H*-gates and *X*-gates each require $\sim\mathcal{O}\ (\log_2(N))$ computations. However, based on the previous discussions in this section, for the *(n-1)-control-1-NOT* gate we require $2((n-1)-1)CCX$ gates, which leads to $\sim\mathcal{O}\ (\log_2(N))$ gates. Thus, in general $\#ofQ_{diff.}\sim\mathcal{O}\ (\log_2(N))$.

The total cost of a quantum algorithm will be calculated as, $\#ofQ_{tot} = \#ofQ_{init.} + \sqrt{N} \times (\#ofQ_{orcl.} + \#ofQ_{diff.})$. Using the discussions in this section, the $\#ofQ_{tot}$ will require
$\sim\mathcal{O}\ [(\log_2(N)) + \sqrt{N} \times \{[\log_2(\log_2(N)) \times ((\log_2(N))^2 + \log_2(N) + 1)] + \log_2(N)\}]$ gates. (Fig. S6).

**II: classic circuit**

Since we do not have any information on implementing the energies in the classical circuits, we ignore its cost in our calculations. Thus, only the adder circuit cost remains. In a classic algorithm, the circuits require adders to add the energy values provided in the energy tables for each interaction. Using a similar-to-quantum adder requires $\sim\mathcal{O}(m) \approx \mathcal{O}(\log_2(\log_2(N)))$ computations. Applying running it for each interaction *i*, requires $\sim\mathcal{O}(\log_2(N))$ computation. Thus, the total cost of an adder circuit, and in general the cost of computation for classic circuit ($\#ofC$), is $\sim\mathcal{O}(\log_2(\log_2(N)) \times \log_2(N))$, similar to

quantum circuits. In addition, there is a cost of subtracting the $E_{tot}$ from the $E_{th}$, which adds another $\sim\mathcal{O}(\log_2(\log_2(N)))$.

The total number of computations for classic algorithm is $\#ofC_{tot} = \mathcal{O}(N) \times (\#ofC)$, which is simplified as $\sim\mathcal{O}(N \times [(\log_2(\log_2(N)) \times \log_2(N)) + \log_2(\log_2(N))])$, which could be simplified as $\sim\mathcal{O}(N \times [\log_2(\log_2(N)) \times (\log_2(N) + 1)])$ (Fig. S6)

## III: comparing quantum and classic

Based on the data provided in Fig. S6, we can see that from the small N values (i.e., N>56), the number of computations in the classic realm surpasses the quantum realm. The smallest system we have for the SP model with *s=2* has *n=6* and *N=64* showing that even for the smallest system the quantum algorithm has lower quantum computation.

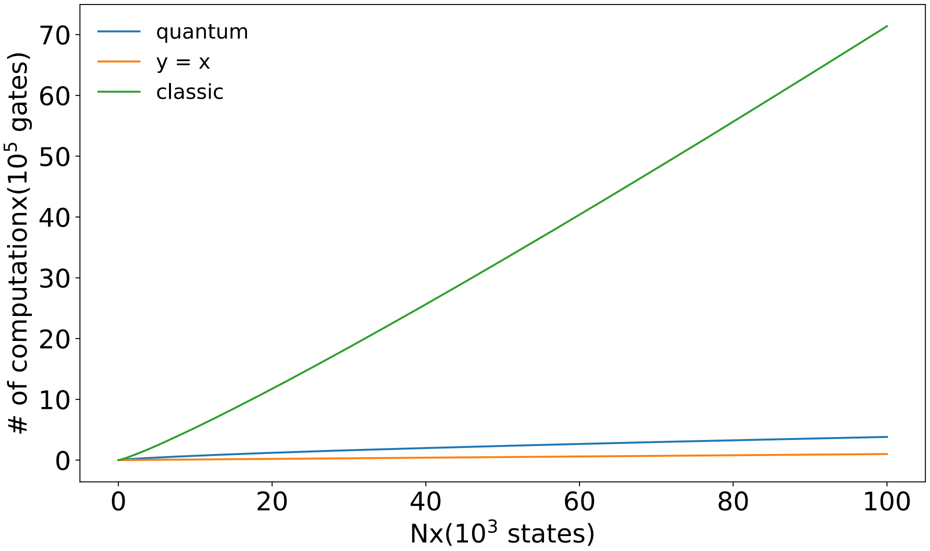


**FIG. S6**. Comparison between the number of gates for classic and quantum algorithms.

## III: MR model

For the MR model, the $\#ofQ_{init.}$ and the $\#ofQ_{diff.}$ are the same as the SP model. Even in the $\#ofQ_{orcl.}$ the introducing the energy, subtracting the $E_{tot}$ from $E_{th}$ and the negation steps are identical to the SP model. The only difference is that for the SP model the energy values in the energy table are simply added to find the $E_{tot}$, while in the MR model, these values are multiplied with the $d^{-1}$. Since the same procedure with the same computational cost does occurs in the classical version of the MR model, the general computational cost will still be much higher for the classical model, compared to the quantum MR model.
