# Supplementary material for: Gate-based quantum computing for protein design
Source: PLoS Comput Biol. 2023 Apr 12;19(4):e1011033. doi: 10.1371/journal.pcbi.1011033 (PMC10124842; doi:10.1371/journal.pcbi.1011033)
Supplement: S6 Appendix — (PDF) [file pcbi.1011033.s006.pdf]

S6. IMPLEMENTING ENERGIES IN THE CIRCUIT

In this part, we show parts of a circuit where some pair-wise energies are implemented using quantum gates (Fig. S7). Here, we have two interacting residues (Fig. S7-A), with the energy table described in Fig. S7-B. Since we have two designable sites, we require *n = 6* qubits, where each residue uses 3 qubits as shown in Fig. S7-C. In the circuit shown in Fig. S7-C, parts of the energy table are implemented through *multi–control–not gates*, specifically *6-control-1-NOT* gates.

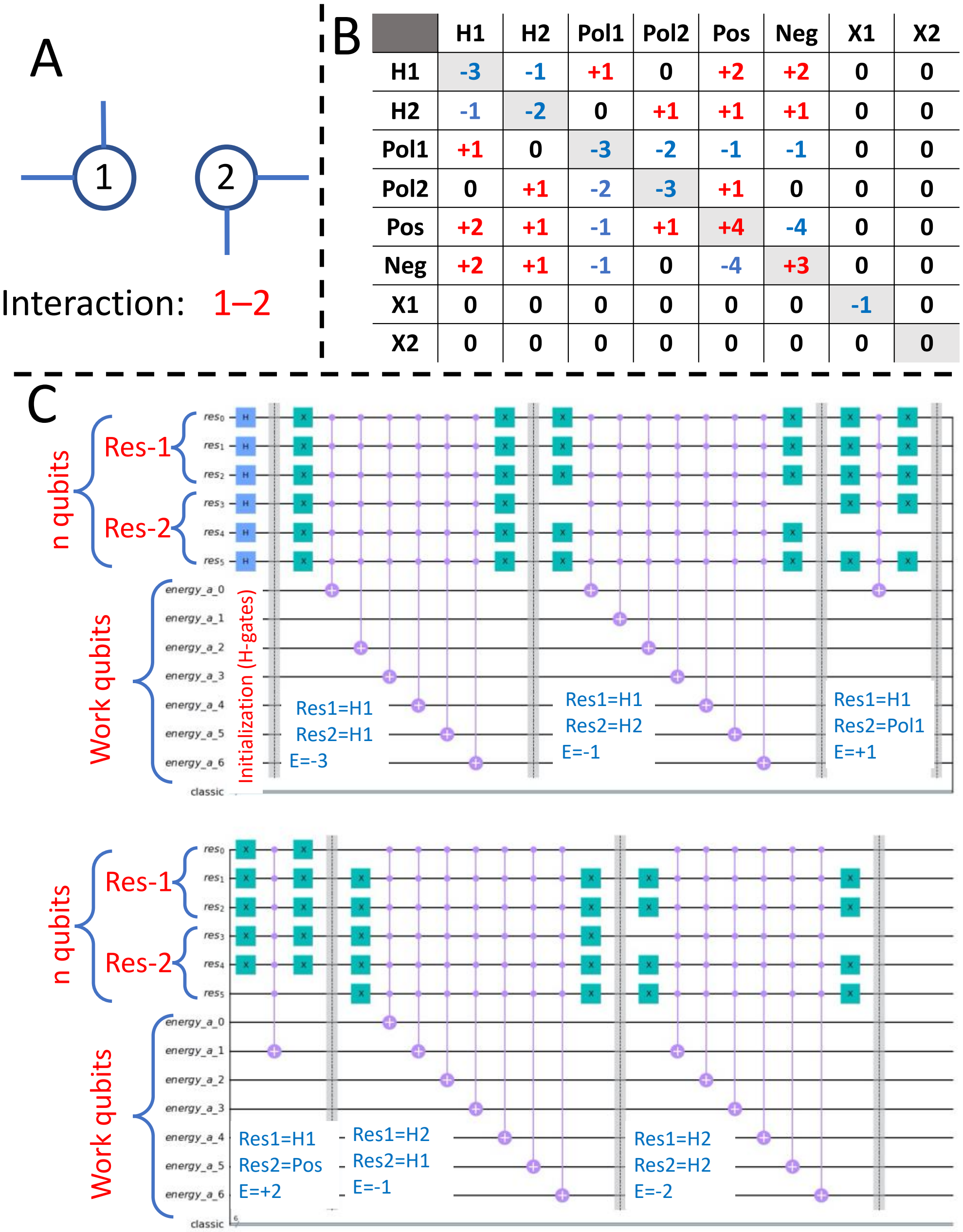

	H1	H2	Pol1	Pol2	Pos	Neg	X1	X2
H1	-3	-1	+1	0	+2	+2	0	0
H2	-1	-2	0	+1	+1	+1	0	0
Pol1	+1	0	-3	-2	-1	-1	0	0
Pol2	0	+1	-2	-3	+1	0	0	0
Pos	+2	+1	-1	+1	+4	-4	0	0
Neg	+2	+1	-1	0	-4	+3	0	0
X1	0	0	0	0	0	0	-1	0
X2	0	0	0	0	0	0	0	0

FIG. S7. A) representation of two interacting residues. B) energy table used in our study. C) Circuit representation of the initialization step and implementation of some of the pair-wise energies in the circuit, with *g=3*, *n=6* and *m=7*.
