# Supplementary material for: Gate-based quantum computing for protein design
Source: PLoS Comput Biol. 2023 Apr 12;19(4):e1011033. doi: 10.1371/journal.pcbi.1011033 (PMC10124842; doi:10.1371/journal.pcbi.1011033)
Supplement: S7 Appendix — (PDF) [file pcbi.1011033.s007.pdf]

S7. Computational Cost of Simulations and Circuit Depth

Despite obtaining the quantum advantage expected from Grover's algorithm (Fig. 6 in the main text), since we use quantum computer simulators to study these systems, running our circuits are restricted by the limitations of the conventional computers, i.e., the amount of RAM required to simulate a system and how long the simulation will take in real-time (CPU time). The changes in the amount of RAM and CPU time required to simulate different systems as a function of R show a linear increase for both the RAM usage and the CPU time for all data sets, which are provided in Supplementary section S-III. This linear increase is expected as by adding the number of iterations, the number of times the oracle and the diffuser are called in the algorithm are increased (Fig. 1 in the main text). Thus, since the simulations run on conventional computational resources, increasing the amount of computation linearly with R increases the cost of computation linearly.

Our results show that simpler circuits, i.e., $s=2$ and $s=3$ in the SP model and $s=2$ in the MR model, could run with $R=R_{max}$ on resources available on a regular laptop device. For these systems, the required RAM amount is ~8 GB, and the maximum CPU time is within an hour time range. However, for the rest of the circuits, simulating only the first few iterations could take days of simulation and more than 10 GB of RAM. For example, the $s=6,i=5,E_{th}=-19$ system would need ~1.5 TB of RAM and ~71,392 hours (~8.1 years) of CPU time to simulate with $R = R_{max}=284$ iterations. Moreover, for systems with the same number of designable sites and interactions, the computational resources required to simulate the circuits are almost identical for all E_{th} values, which indicates that the usage of computational resources is independent of the E_{th}.

Comparing the results between systems with similar configurations and results in the SP and MR model, i.e., the $s=2,E_{th}=-3$ and the $s=2,E_{th}=95\%E_{min}$ circuits, show that the CPU time is more than 18 times longer and the RAM usage is twice more in the MR model circuits. These differences are expected as increasing the complexity of the oracle in the circuit increases the computational cost of simulations.

Figure S.8 provides insight into the depth of the quantum circuits and their effect on the CPU time and the RAM usage of simulations. Our results show that by increasing the circuit depth, the RAM usage and the CPU time for all simulations increase with similar but distinct power-law behaviours (Fig. S.8-a and b). This similarity confirms the consistency of circuits and gates among systems in each model. However, the different types of gate-based calculations performed in the oracle for each model (and the number of qubits required to execute them) lead to the distinct behaviour of the RAM usage and CPU time patterns in the SP and MR models. Moreover, the increase in the CPU time is faster for simulations in the MR model, while the RAM usage grows faster for systems in

the SP model, which could be related to the way the simulator is programmed and is beyond the scope of this study.

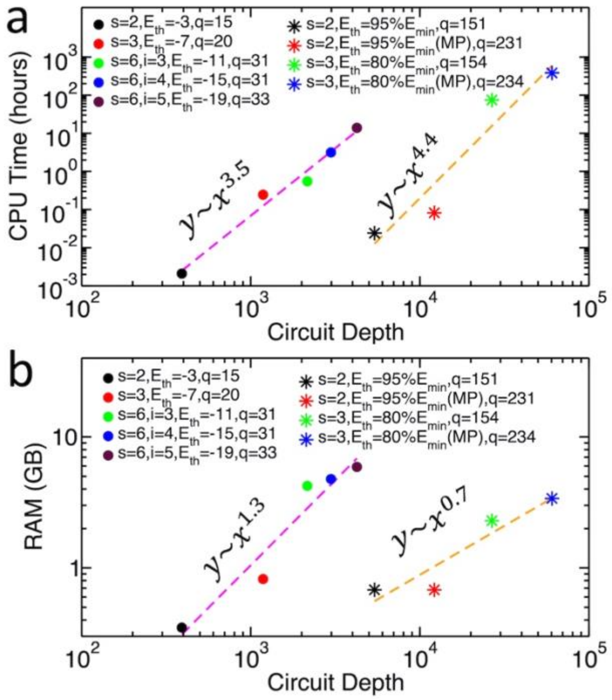

FIG. S8: Role of circuit depth in computational costs of simulating the circuits at R=1. Change in the: a) RAM usage; b) CPU time as a function of circuit depth. The dashed lines represent the fit to the data points. The q indicates the total number of qubits in the circuit.
