# Supplementary material for: Gate-based quantum computing for protein design
Source: PLoS Comput Biol. 2023 Apr 12;19(4):e1011033. doi: 10.1371/journal.pcbi.1011033 (PMC10124842; doi:10.1371/journal.pcbi.1011033)
Supplement: Attachment — Submitted filename: QC_Paper_answer.docx [file pcbi.1011033.s009.docx]

Editors

PLoS Computational Biology

Toronto, March 13, 2023

**Re: PLoS Computational Biology submission EMID:3a9387d6b6711d10**

Dear Dr. Krems and. Ben-Tal,

We appreciate the time and energy that the editors and reviewers have dedicated to reviewing our manuscript. We have provided our answers to the comments of reviewers and changed the manuscript accordingly, as suggested by the reviewers. Moreover, the code we have used to generate the results of this paper are provided through GitHub and properly cited in the current version of the manuscript. Below, we have provided the original comments and the answers we have provided. The answers to the comments are in blue, and the changes to the manuscript text are in green.

With best regards,

Philip M. Kim, Ph.D.

Reviewer #1:

I have read the submission by Khatami, Mendes, Wiebe, and Kim titled “Gate-based quantum computing for protein design”. In this manuscript, the authors examine the optimization problem of finding an amino acid sequence corresponding to the minimum value of an energy or scoring function, a problem that is solved during the protein design process. This problem scales poorly with the number of amino acid positions being designed in the protein (which the authors represent as s), or with the number of amino acid types considered at each position (which the authors represent as A), and this represents a serious challenge for design algorithms on classical computers. The authors show that this problem can be solved on gate-based quantum computers using Grover’s algorithm, a method for searching a large solution space using quadratically fewer samples than would be required classically. Using quantum simulators run on classical hardware, they show that they are able to produce solution states with high probability (quantum computing is probabilistic computing) for small problems, and that noise hinders but does not eliminate the functionality of their approach. They also show enrichment of the solution state for a very small problem on an actual, current-generation IBM gate-based quantum computer.

The authors have identified a sensible problem for which quantum computers could plausibly offer an advantage over classical computers one day. Their approach appears to be sound. The manuscript reads well, like one that has had quite a bit of thought go into revisions already. I am largely satisfied that this manuscript is suitable for publication. Below, I list one concern that I hope the authors can address or rebut, as well as a small number of minor points related to presentation which I think the authors will be able to address easily.

The authors would like to appreciate the positive comments provided by reviewer #1 on the topic of the paper and the manuscript quality.

Major concern:

1. The PLoS author guidelines state: “PLOS expects researchers to share software and scripts needed for the work. If this cannot be made publicly available (e.g. due to licenses), the simulation method should be provided in sufficient detail so the results can, in principle, be reproduced using publicly available software.” I think that the authors have made a good faith effort to describe their methods in a good deal of detail. I am moderately confident that a person with sufficient domain expertise could reproduce what was done here, though the complexity of the approach does mean that it would be hard to say for certain whether any differences that that person saw between their results and the authors’ results was due to errors in reimplementing the method versus actual failure of the method to reproduce the result. I would be more comfortable if the authors were willing to make their Qiskit code for simulating these circuits (or, at least, for simulating a particular problem) publicly available. I won’t insist on this, since the description in the supplement is quite detailed, but it would make me more comfortable for the sake of full reproducibility.

We acknowledge the concern of the reviewer and provide the Qiskit codes used in this study publicly through GitHub. The following text is also added at the end of the manuscript before the References section (lines 665-667).

**Data availability**

All codes for the MR and SP models in this study are available through GitHub with the identifier (https://zenodo.org/record/7344649)[42]**.**

Minor points:

1. On lines 70-72, the statement of the main characteristic of NP-complete problems could be a bit more precise and a bit clearer. The main characteristic of these problems is that the time or resources needed to find a solution scales poorly, not that they are intrinsically costly across all problem sizes. (There are NP-complete problems, like the travelling salesman problem, that are solved routinely for small problem instances, such as getting delivery vehicles to customers.) Similarly, on line 73, the proposed answer need not be scored easily, but in polynomial time. (There are polynomial-time tasks, such as the two-body molecular docking problem, that are extremely costly.) I mention this mainly because this is often a misconception that I have to dispel with students, that NP-completeness intrinsically means computational intractability or that polynomial-time scaling intrinsically means computational tractability for all problem sizes. It’s actually something that’s kind of interesting about the protein design problem: it’s an NP-complete problem with interesting real-world applications across a range of scales, ranging from extremely tractable (allowing classical and quantum solvers to be compared on these problems) to challenging (meaning that there may be problems for which there’s a slight quantum advantage) to utterly intractable (meaning that a good quantum solver would have problems that it was uniquely suited to do).

We appreciate the detailed comment and have applied the suggested comments in the manuscript. Here is the updated text (lines 71-76):

The main characteristics of these problems are that the computational time and resources needed to find their solutions scale poorly. Thus, finding their answer using conventional computers could become either impossible or requires a great deal of computational time. However, the validity of a proposed answer could be evaluated in polynomial time by conventional computers[3].

2. In the caption to Figure 1, there’s a sentence that seems to be incomplete. (As an aside, I like the detail in figure 1 – particularly panel B. Explanations like this in plan language make these algorithms much easier to understand.)

We appreciate the kind compliments on Figure 1. The incomplete sentence is removed.

3. On lines 137-138, “is not advantageous” is a little bit confusing. Maybe it could be made clearer that the sign flip does not, at this point, alter the relative probabilities of states (something that will be altered by the subsequent diffuser step)?

We agree with the reviewer and have modified the text to be clearer (lines 164-165).

… the negative amplitude of the answer states in the oracle step does not change the relative probabilities of states…

4. Figure 2 is pretty clear. A suggestion that the authors might consider is adding a dashed line for each pairwise interaction, maybe in the same dark red colour as the listed interactions. (The lists currently refer to a feature that the reader can infer, but which is invisible. I wonder if it might be a little easier on the reader just to make it more visible.)

We have applied the suggested changes to Figure 2 and updated the caption accordingly (line 170).

… The red dashed lines represent the interactions among the designable sites…

5. On lines 194 and 201, “number of residues” is a bit ambiguous: it could be the number of designable positions, s, or the number of amino acid possibilities at each position, A. I think the latter is meant. Perhaps using the nomenclature established earlier (A) or indicating that this is number of residue types might make this clearer.

We agree with the reviewer and have applied the required changes. Here is the modified text (lines 225-229):

The number of qubits required to represent a residue in a unique binary format is given by $g=\left\lceil{log}_{2}(A) \right\rceil$. Thus, g=3 for the eight amino acids used in both SP and MR models (Fig. 3-a, b and c). Even though our energy tables with eight types of residues are simpler than the canonical model with A=20 (g=5), they are still more complicated than the widely used HP table in the IBM-SP model (g=1).

6. A small singular/plural typo on line 228: “simulate circuits with this many qubits.”

The change is applied.

7. Figures 5 and 6 show good results. The one minor criticism I have is of the x-axis label in figure 5 and the x- and y-axis labels in figure 6: descriptive labels such as “Max number of iterations (Rmax)” are easier to follow than the symbol alone (especially when the axis label is otherwise an expression of several symbols, such as R/Rmax. It’s not always intuitive to a reader why a particular expression is on an axis or what the expected relationship between an abstract expression on one axis and an abstract expression on another should be.)

The suggested changes are applied to the x- and y-axis labels in figures 5 and 6.

8. It’s worth being aware that a circuit depth of 20 (line 415) sounds a little bit low as an upper limit. My experience has been that circuit depth limits of 40 or 50 seem a little more realistic, currently. This is very minor, though: either way, the authors’ point that only very short circuits are currently possible is a valid one.

We appreciate the comment. After a more detailed search, we found an article that we had initially missed on the depth of the circuits, mentioning the depth of ~50, as suggested by the reviewer. The text in the manuscript is changed accordingly (lines 466-468).

… As Chia *et al.* [35] and Li *et al.* [36] have discussed, circuits that could run on currently available real quantum devices are mostly limited to the circuit depth of only a few tens of gates, with a maximum of ~55. These studies suggest …

9. On line 444, “Coulomb” should be capitalized.

The change is applied (line 499).

10. I’d be a little bit careful with the claim on lines 446-448, that the distance reciprocal and decimal number approach used here could be used in lattice model folding studies. If the claim is that one could keep the lattice model, but use pairwise interaction energies represented as decimal numbers, possibly taken from classically precomputed lookup tables, this sounds reasonable. If the claim is that this provides a route to non-integer positions for the beads on the chain (i.e. a means of moving past lattice models to continuous-space models), this is harder to envisage, and would require means of dealing with very non-pairwise effects (such as the fact that the position of residue 3 depends on both residue 1 and residue 2). Maybe rephrasing this to make it clear that the former is meant would be a good idea.

We agree with the comment and have updated the text accordingly to clarify our points (lines 501-504).

Moreover, our approach in implementing the pre-calculated distances reciprocals and decimal numbers and using multiplication functions could also be employed in the protein folding studies with quantum computers currently limited to uniform lattice models[11–14].

11. In the first paragraph of the methods, it might be good to make it even more explicit what’s done classically and what’s done in the quantum circuit. For instance, “First, the classically-computed values in the energy tables are introduced to the oracle… Next the energy values for every pair-wise interaction in the structure are summed in the quantum circuit to find the total energy…”

The suggested changes are applied to the manuscript (lines 526-530):

… First, the classically-calculated values in the energy tables (Fig. 3)…

… Next, the energy values for every pair-wise interaction in the structure are summed using quantum computing adders to find the total energy…

12. The point made on lines 549-555, about the circuit depth needed for a real problem compared to what’s currently possible, is an important one, and one that many readers will be looking for in the conclusions. It’s likely worth including in the discussion in more detail, not just in the methods. I suspect that many readers will be looking for this as a bottom line to the story: they’ll be wondering, “at what point is the quantum hardware likely to be useful for real design problems”? (Note: I do NOT consider the fact that real problems will require larger, more robust hardware than is currently available to be a shortcoming of this work.)

Thank you for the comment. the following text is added to the discussion section (lines 506-515):

… with using each gate limit studying pure quantum algorithms on real quantum devices. In these devices, since the depth of circuits is restricted to a few tens of gates, even running a small protein design circuit (i.e., IBM-SP) does not provide a definitive result due to the noise and requires further improvements in quantum computers. Moreover, for the SP,s=2 system, the depth of the circuit in a fully connected simulator is ~400 gates, while after transpilation, the depth of the circuit on real quantum devices could reach ~17,000 gates. As an alternative approach….

Reviewer #2: Summary:

This paper proposes to use Grover’s algorithm to solve the protein design problem, which asks to decide the amino acid type on each designable site to achieve the lowest energy. In contrast to prior gate-based hybrid quantum-classical computation and quantum annealing, this work presents a gate-based pure quantum algorithm solution. The main task in this work is to construct the oracle in Grover’s algorithm. With the oracle, Grover’s algorithm can be applied to find the desired solution. It shows that the expected quadratic speed-up can be achieved compared to classical brute-force search. Experimental justification is performed on simulators and real quantum devices to show the correctness of the method.

We appreciate the comments reviewer#2 has on highlighting the points on the strengths and weaknesses of our work.

Strengths:

- The protein design problem is important and its effective solutions will be widely useful.
- The first gate-based pure quantum algorithm for the protein design problem is proposed.
- The method is flexible in customizing for different energy tables and protein structure models.
- Related work is adequately cited.

Weaknesses:

- The design of the oracle is somewhat brute-forced, leading to extremely high costs.

It should be considered that this is the first try at using Grover’s algorithm for protein design. The main point of our work is to show if this approach works. In the next step, if the method we have used is somewhat universal, i.e., one can implement different numbers of designable sites with different energy tables and model complexities (SP and MR) with fairly simple modifications in the oracle. In the next phase of developing this approach, we plan to work on possible improvements in our oracles.

- The oracle construction, which is the main technicality of the paper, is not well described.

We have worked on explaining the details of the algorithm and the different steps of creating the oracle in the main text and supplementary data. Moreover, we provide the codes to the SP and MR models used in this study to solve this point in the revised version of our manuscript.

- There is no experimental comparison with prior methods.

As discussed in the introduction section of the manuscript, the main point of our research is that there are no previous works on using gate-based pure quantum computing algorithms for protein design.

Specific comments:

Grover’s algorithm is a well-known quantum algorithm. Therefore, the main contribution of this work is designing the oracle to make the algorithm applicable to the considered problem of protein design. However, the article does not focus on explaining the oracle construction, but rather spends too much effort introducing Grover’s algorithm. Moreover, the oracle seems to directly use existing structures of adders and multipliers, trying to do numerical operations directly on quantum circuits. This direct adoption without optimization leads to extremely high costs, limiting the problem to small examples (e.g., 6 designable sites) even for simulation-based justification. It would be helpful if optimization can be performed to minimize the oracle circuit.

Thank you for your comments. We agree that our paper tries to explain Grover’s algorithm in more detail. It is an intentional effort as the bulk of the computational biology society members are less familiar with quantum computing, specifically Grover’s algorithm. Moreover, current quantum computing research, mainly in algorithm development, is focused on classic/quantum hybrid variational approaches. Thus, a detailed explanation of Grover’s algorithm is deemed necessary.

Regarding the less attention to explaining the oracle details, we want to point out that lines 527 to 588 (in the new version of the manuscript) and sections S-I and S-VI in the supplementary data are dedicated to describing details of our oracles. However, we have also included the codes for the circuits used in this study in the revised version of the manuscript.

Regarding the efficiency of our algorithms, It should be considered that this is the first try at using Grover’s algorithm for protein design. The main point of our work is to show if this approach works. In the next step, if the method we have used is somewhat universal, i.e., one can implement different numbers of designable sites with different energy tables and model complexities (SP and MR) with fairly simple modifications in the oracle. In the next phase of developing this approach, we plan to work on possible improvements in our oracles. Moreover, current examples of implementing Grover’s algorithm are limited to simple problems that are mentioned in our introduction section. Thus, we are confident that our manuscript will contribute to implementing Grover’s algorithm (and generally pure quantum algorithms) in real-life applications.

The value of E_th may seem a heuristic coming from an empirical experience. Although Eq. (6) and Eq. (7) may result in a good performance (providing few answer states) in the tested benchmarks, their applicability and generality may be questionable. For example, if there are more designable sites, maybe 95% E_min may be unattainable for any candidate states, leading to empty solution space. It would be good to discuss how to set E_th. Moreover, it seems that this method only finds solutions with lower energy rather than the lowest energy.

We appreciate reviewer #2 comment on choosing E_th values in the SP and MR models. The E_th values for the SP models (calculated using Eq.6) indeed represent the lowest energy to find the lowest energy systems in our study. However, as reviewer #2 has correctly mentioned, the E_th for the MR model, calculated using Eq.7, shows the lowest limit below which there would definitely be no answer states (at B=1). That is why we have stated that B is less than 1. However, as shown implicitly in Table S-2 in the supplementary data (S.II), by increasing the number of designable sites, the B value decreases from ~0.95 in the s=2 system to ~0.85 in the s=3 system to find the possible lowest energy states. Note that for the MR model, in addition to the number of designable sites, the values of distance reciprocals play an important role in the value of E_th to find the lowest energy state.

We have added the following text to further clarify the E_th for SP and MR models (line 571-580).

Moreover, it should be noted that the Eth value calculated for the SP model system (Eq.6) captures the lowest energy structures for different numbers of designable sites (see results in Supplementary section S-II). However, the Eth value calculated for the MR model (Eq.7) does show the limit below which there are no answer states (for B=1). Due to the presence of distance reciprocals and the fact that all designable sites interact in the MR model circuits, one should carefully set the B value to attain the desired results. As the number of designable sites increases, the B value and, equivalently, the Eth value to find the state with the lowest energy decreases (see results in Supplementary section S-II for the MR,s=2 and MR,s=3 systems). Unlike the SP model, the Eth in the MR model does not define the lowest energy states but a heuristic threshold from an empirical experience to find low energy states.

Although related work is introduced, a more in-depth comparison should be performed to reveal the strengths and weaknesses of different approaches.

We agree with reviewer #2 that comparing the results between different approaches should be conducted to show the strength of one approach over another. However, we want to point out that no prior studies have used pure quantum computing algorithms for protein design, and our approach is the first one.
